# Effects of pharmaceutical formulations containing thyme on carbon tetrachloride-induced liver injury in rats

**DOI:** 10.1186/s12906-015-0966-z

**Published:** 2015-12-18

**Authors:** Aleksandar Rašković, Nebojša Pavlović, Maja Kvrgić, Jan Sudji, Gorana Mitić, Ivan Čapo, Momir Mikov

**Affiliations:** Department of Pharmacology, Toxicology and Clinical Pharmacology, Faculty of Medicine, University of Novi Sad, Hajduk Veljkova 3, Novi Sad, 21000 Serbia; Pharmacy “Novi Sad”, Rumenačka 1, Novi Sad, 21000 Serbia; Institute of Occupational Health, Futoška 121, Novi Sad, 21000 Serbia; Institute of Laboratory Diagnostics, Clinical Center of Vojvodina, Hajduk Veljkova 1, Novi Sad, 21000 Serbia; Department of Histology and Embryology, Faculty of Medicine, University of Novi Sad, Hajduk Veljkova 3, Novi Sad, 21000 Serbia

**Keywords:** Thyme, Hepatotoxicity, Oxidative stress, Antioxidant enzymes, Tincture, Thymol

## Abstract

**Background:**

Herbal supplements are widely used in the treatment of various liver disases, but some of them may also induce liver injuries. Regarding the infuence of thyme and its constituents on the liver, conflicting results have been reported in the literature. The objective of this study was to examine the influence of two commonly used pharmaceutical formulations containing thyme (*Thymus vulgaris L.*), tincture and syrup, on carbon tetrachloride-induced acute liver injury in rats.

**Methods:**

Chemical composition of investigated formulations of thyme was determined by gas chromatography and mass spectrometry. Activities of enzyme markers of hepatocellular damage in serum and antioxidant enzymes in the liver homogenates were measured using the kinetic spectrophotometric methods. Liver morphology was characterized by light microscopy using routine hematoxylin and eosin staining.

**Results:**

Thymol was found to be predominant active constituent in both tincture and syrup. Investigated thyme preparations exerted antioxidant effects in liver by preventing carbon tetrachloride-induced increase of lipid peroxidation. Furthermore, co-treatment with thyme preparations reversed the activities of oxidative stress-related enzymes xanthine oxidase, catalase, peroxidase, glutathione peroxidase and glutathione reductase, towards normal values in the liver. Hepatotoxicity induced by carbon tetrachloride was reflected by a marked elevation of AST and ALT activities, and histopathologic alterations. Co-administration of thyme tincture resulted in unexpected exacerbation of AST and ALT values in serum, while thyme syrup managed to reduce activites of aminotransferases, in comparison to carbon tetrachloride-treated animals.

**Conclusions:**

Despite demonstrated antioxidant activity, mediated through both direct free radical scavenging and activation of antioxidant defense mechanisms, thyme preparations could not ameliorate liver injury in rats. Molecular mechanisms of diverse effects of thyme preparations on chemical-induced hepatotoxicity should be more in-depth investigated.

## Background

Herbal medicinal products are increasingly being used and many of them have shown promising potential for the treatment of various diseases. However, many of herbal medicines remain untested and their use also not monitored, which makes knowledge of their safety very limited [1]. Besides, herbal medicines are widely promoted as being ‘natural’, and therefore are perceived to be ‘safe’, which is not only untrue, but also misleading [2].

The liver is highly susceptible to drug-induced toxicity due to its significant role primarily in drug metabolism. The pathophysiological mechanisms of drug-induced hepatotoxicity are still to be elucidated, but are mostly initiated by generation of hepatotoxic metabolites in the phase I of drug metabolism that often leads to the mitochondrial inhibition and accumulation of reactive oxygen species (ROS). Oxidative stress in hepatocytes finally results in apoptosis and necrosis of cells [3, 4]. Considering the role of oxidative stress in pathogenesis of a wide range of liver diseases, natural antioxidant products, especially phytochemicals, are widely used in their treatment and nearly half of the agents used in liver therapy today are either natural products or their derivatives [5]. On the other hand, the incidence of liver injuries due to herbal and dietary supplements have risen largely in the past decade, probably in part due to their growing consumption [2]. Thus, it is necessary to carefully determine the influence of various herbal medicines on liver function, in both normal and pathological conditions.

Thyme (*Thymus vulgaris* L., Lamiaceae), a small subshrub native to the western Mediterranean region of Europe, is commonly used as a culinary herb, but also for different medicinal purposes. Antitussive, expectorant and spasmolytic effects are considered to be the major pharmacological properties of thyme, and therefore it is used for diseases of the upper respiratory tract, alone or in combination with other herbs [6]. The German Commission E approved internal use of thyme preparations for treating symptoms of bronchitis, whooping cough and catarrh of the upper respiratory tract [7]. Additional therapeutic properties of thyme include antioxidant [8] and antimicrobial activity [9], genotoxic [10], anti-inflammatory [11], analgesic and antipyretic effects [12], and antidiabetic effects [13], among others. Several thyme preparations, such as dried herb, liquid extract, elixir, and tincture, are included in different official monographs, and they are commonly added to formulations involving multiple herbal constituents, most often to syrups, but may be also incorporated in tablets [14].

The main uses of thyme in culinary and food processing are defined by its odour and taste, but also antioxidant and antimicrobial activities. Food flavouring still remains the main thyme application area in culinary, while its antimicrobial and antioxidant properties can be considered as the supplementary benefits of thyme products, which have been added to the foods [15].

Most pharmacological effects of thyme are the consequence of its high antioxidant activity that is mainly attributed to the presence of phenolic monoterpenes, thymol and carvacrol, as the major constituents of essential oil of thyme. On the other hand, phenolic acid (rosmarinic acid) and flavonoids (quercetin, eriocitrin, luteolin, and apigenin) are proposed to be the polyphenolic compounds responsible for the antioxidant effects of ethanolic and aqueous extracts [16]. It should be noted that many plant species, including thyme, contain individuals with distinct composition of the secondary metabolites, i.e. chemical phenotypes (chemotypes). Regarding the essential oils, seven chemotypes have been described within Thymus species, defined by the single dominant monoterpene produced: thymol, carvacrol, geraniol, α-terpineol, sabinene hydrate, linalool, or 1,8-cineole [17]. Therefore, all effects of thyme preparations should be carefully examined, taking into account the chemical composition.

Regarding the infuence of thyme and its constituents on the liver, conflicting results have been reported in the literature. Although thyme has been listed as Generally Recognized as Safe (GRAS) for use in food, it is known that long-term or high-dose administration of thyme preparations such as tinctures and essential oils that contain thymol and carvacrol in high amounts, can be hepatotoxic [18]. Use of these products may aggravate existing liver damage in humans [19]. On the contrary, the hepatoprotective effects of thyme have been observed in several experimental models of liver injury. The ethanolic and methanolic extracts of thyme have been effective against aflatoxins- and N-nitrosodiethylamine (NDEA)-induced oxidative liver damage, respectively [20, 21]. Thyme extract and essential oil could ameliorate carbon tetrachloride (CCl_4_)-induced liver injury in rats [22]. The protective effects of aqueous extract [23] and essential oil obtained from thyme [24] have been also demonstrated in experimental model of paracetamol-induced liver damage.

Therefore, the objective of our study was to examine the influence of two pharmaceutical formulations containing thyme, tincture and syrup, which are commonly used in humans, on CCl_4_-induced acute liver injury in rats.

## Methods

### Thyme formulations and chemicals

Pharmaceutical formulations of thyme (*Thymus vulgaris* L., Lamiaceae) used in this study were tincture and syrup. They were obtained from the Institute for Studies on Medicinal Plants ’Dr Josif Pančić’, Belgrade, Serbia, in 2012. A voucher specimen of the plant (*Thymus vulgaris* L. 1753 subsp. *vulgaris* N^o^ 2–1516, det.: Goran Anačkov) was confirmed and deposited in the Herbarium of the Department of Biology and Ecology, Faculty of Sciences, University of Novi Sad [25]. The tincture was made from the aerial parts of the plant (*Thymi herba*) with 70 % ethanol in a 1:5 ratio of weight to volume. Thyme syrup consists of thyme tincture (15 % w/w) and potassium bromide, simple syrup, methyl and propyl p-hydroxybenzoate as preservatives, and water.

Carbon tetrachloride was obtained from Merck (Darmstadt, Germany). All chemicals for biochemical assays were purchased from Sigma-Aldrich (St Louis, MO, USA).

### Qualitative and quantitative analysis of thyme tincture and syrup

The identification and quantification of thymol and carvacrol, as main chemical constituents of thyme tincture and syrup, were carried out by gas chromatography coupled with mass spectrometric detection (GC/MS) and flame ionization detection (GC/FID). GC/MS analysis was performed using an Agilent 7890A GC system equipped with 5975C VL MSD (Agilent Technologies, CA, USA). The capillary column used in this study was DB-5MS (30 m × 0.25 mm, film thickness of 0.25 μm; J&W Scientific, CA, USA). The temperature program was set as follows: initial temperature 50 °C held for 1 min, 5 °C per min to 100 °C, 9 °C per min to 200 °C held for 7.89 min, and the total run time was 30 min. The flow rate of helium as a carrier gas was 0.811851 ml/min. The MS system was operated in electron ionization (EI) mode with selected ion monitoring (SIM). The ion source temperature and quadrupole temperature were set at 230 °C and 150 °C, respectively. Identification of thymol and carvacrol was performed by comparison of their retention times and mass spectra with those of authentic standards and with spectra in the NIST08.L and Wiley7n.l libraries.

GC/FID analysis was carried out using a Hewlett Packard 6890 chromatograph equipped with flame ionization detector, autosampler and a split/splitless injection system. The column DB-5MS (60 m × 0.32 mm, film thickness of 0.25 μm; J&W Scientific, CA, USA) and helium as a carrier gas with flow rate of 1.9 ml/min were used in this analysis. Samples in volumes of 1 μl were injected in a split mode (split ratio, 2.5:1). The injector and detector temperatures were set at 250 °C and 300 °C, respectively, and the column temperature program was set as follows: initial temperature 60 °C held for 1 min, 10 °C per min to 265 °C held for 8.5 min, with the total run time of 30 min. The concentrations of thymol and carvacrol were determined using the calibration curves prepared with standards of thymol and carvacrol, and cumene as internal standard.

### Evaluation of *in vitro* antioxidant activity and total phenolic content of thyme tincture

Antioxidant activity of the tincture of thyme was evaluated as free radical scavenging capacity (RSC). The ability of tincture to donate an electron and scavenge the stable 1,1-diphenyl-2-picrylhydrazyl (DPPH) radical was investigated [26]. The tincture of thyme and aqueous solution of ascorbic acid, as a positive control, were mixed with DPPH solution and after 60 min, remaining amount of the purple-colored DPPH radical was measured spectrophotometrically at 515 nm. Free radical scavenging capacity was calculated as follows: RSC = 100–100 * Asample/Ablank, where Ablank is the absorbance of diluted DPPH solution and Asample is the absorbance of the tincture or reference. The IC_50_ value, which represents the concentrations of the sample required to cause 50 % inhibition of DPPH radical, was estimated by linear regression analysis from the obtained RSC values and was expressed in mg/ml.

Phenolic content of tincture was determined using the Folin-Ciocalteu reagent [27]. The tincture was mixed with Folin-Ciocalteu reagent and sodium carbonate solution in test tube. After being vortexed and incubated in dark for 2 h, absorbance was measured at 740 nm. The result was expressed as mg of gallic acid equivalents (GAE) per liter.

### Experimental design

The experiment was performed on 7–8 week old albino Wistar rats of both sexes, weighing 230–300 g, obtained from Military Medical Academy, Belgrade. The rats were housed in standard laboratory cages, under a 12 h light-dark cycle, at a constant ambient temperature (23 °C) and humidity (30–50 %), and were allowed to adapt for 2 weeks before the experiments were started. The animals were maintained on standard pellet diet (LM2, Veterinary Institute, Subotica, Serbia) and allowed access to tap water *ad libitum*. Animal care and all experimental procedures were conducted in accordance with the Guide for the Care and Use of Laboratory Animals edited by Commission of Life Sciences, National Research Council (USA). The study was approved by the Ethical Committee of the University of Novi Sad (Approval No. 01-53/7-1).

To evaluate the effects of thyme formulations on CCl_4_-induced liver injury, the animals were randomly divided into six experimental groups, each containing six individuals, and treated as follows:

- Con S: Control group, saline 1 ml/kg *p.o.*
- Con CCl_4_: saline 1 ml/kg *p.o.* + a single CCl_4_ dose 1 ml/kg *i.p.*
- Tin: thyme tincture 0.18 ml/kg *p.o.*
- Tin + CCl_4_: thyme tincture 0.18 ml/kg *p.o.* + a single CCl_4_ dose 1 ml/kg *i.p.*
- Syr: thyme syrup 5.6 ml/kg *p.o.*
- Syr + CCl_4_: thyme syrup 5.6 ml/kg *p.o.* + a single CCl_4_ dose 1 ml/kg *i.p.*

Applied daily doses of thyme tincture and syrup for rats were 0.18 ml/kg and 5.6 ml/kg, respectively. Recommended human daily doses of thyme tincture and syrup for a male of approximately 70 kg weight [28] were adapted for the experimentation on rats using FDA guidance [29]. Thyme preparations were administered every day for seven days by *per os* gavage, previously diluted with saline. On the 7th day, 1 h after the last dose of thyme preparations or saline, animals were treated intraperitoneally with CCl_4_ dissolved in olive oil (1:3, 1 ml/kg) [30]. After 24 h, rats were anesthetized with urethane (0.75 mg/kg) and sacrificed by cardiopunction, and the samples of blood and liver were taken. The obtained serum was used for determination of standard biochemical parameters. Liver homogenates were prepared from 1 g of liver tissues which were homogenized in a Potter homogenizer with Tris-HCl sucrose buffered solution in a ratio 1:3 (w/v) at 4 °C. The parameters of oxidative stress were analyzed in obtained liver homogenates.

### Serum biochemical parameters determination

Total cholesterol and triglycerides levels, concentration of bilirubin, activities of alanine aminotransferase (ALT) and aspartate aminotransferase (AST), and the concentrations of urea and creatinine were determined in serum using commercially available kits based on the well-established spectrophotometric methods, according to the manuals supplied. All analyses were performed in triplicate for every sample on the Olympus AU 400 autoanalyzer (Hamburg, Germany).

### Determination of oxidative status in the liver

Oxidative status in the liver was estimated by measuring the levels of lipid peroxidation (LPx), and the activities of oxidative stress-related enzymes, including catalase (CAT), peroxidase (Px), glutathione peroxidase (GPx), glutathione reductase (GR) and xanthine oxidase (XOD) in liver homogenates, using the spectrophotometric methods.

The intensity of LPx was estimated by measuring the amount of malondialdehyde, a terminal product of lipid breakdown due to peroxidation damage, using the method of Buege and Aust, 1978 [31]. XOD activity determination method was based on the measuring the rate of methylene blue reduction [32]. CAT activity was determined by the method based on monitoring the H_2_O_2_ decomposition rate at 240 nm [33], and Px activity by using guaiacol as the enzyme’s substrate [34]. The activities of GPx and GR were determined following the methods of Beutler, 1984 [35] and Goldberg and Spooner, 1983 [36], respectively. These tests were based on the measuring the decrease in absorbance caused by the oxidation of NADPH at 340 nm (UV assay).

### Histological assessment

Liver morphology was characterized by light microscopy using routine hematoxylin and eosin (H&E) staining. Twenty-four hours after administering CCl_4_, a small piece of hepatic tissue was removed for histological analysis. Liver tissue samples from each rat were fixed in Bouin’s solution for 24 h, then dehydrated in a graded series of isopropyl alcohol, and embedded in paraffin blocks. Five-micrometer tissue sections were cut using a rotation microtome (Leica Microsystems) in triplicate for each animal, and stained with hematoxylin and eosin for histological analysis under Olympus BX-43 light microscope (Olympus, Japan) with an attached Olympus DP 73 video camera (Olympus, Japan).

### Statistical analysis

Data are expressed as mean ± standard error of the mean (SEM). The intergroup variation between various groups was measured by one-way analysis of variance (ANOVA) followed by Tukey’s multiple comparison test. Results were considered statistically significant when *p* < 0.05. Data were analyzed using SPSS software, version 19 (IBM SPSS, Chicago, IL, USA).

## Results

### Concentrations of thymol and carvacrol in thyme preparations

**Fig. 1:**
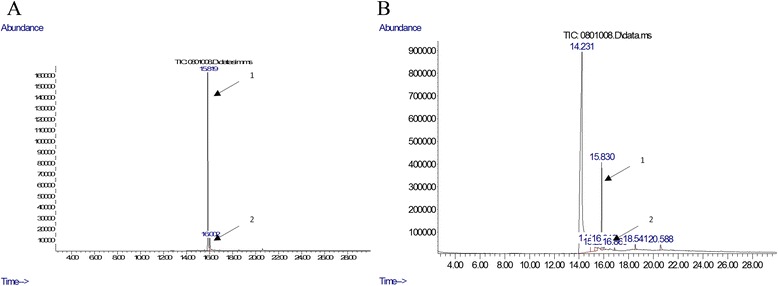
GC/MS chromatograms of thymol and carvacrol standards (**a**) and thyme syrup (**b**)

Thymol and carvacrol, as the major phenolic active compounds of thyme, were identified and quantified in examined thyme formulations by GC/MS. Thymol concentrations of 175.3 μg/ml and 9.73 μg/ml were measured in tincture and syrup, respectively. Concentrations of carvacrol were much lower than those of thymol in both formulations, and they were 10.54 μg/ml and 0.55 μg/ml in tincture and syrup, respectively. GC/MS chromatograms of thymol and carvacrol standards and thyme syrup are shown in Fig. 1. Peaks 1 and 2 were identified as thymol and carvacrol at retention times of 15.819 and 16.002 min, respectively. Monitored ions (m/z values) used for quantification and confirmation, and retention times of thymol and carvacrol in GC/MS analysis are presented in Table 1.

**Table 1:** GC/MS parameters used for determination of thymol and carvacrol in thyme tincture and syrup

Analite	Monitored ions (m/z)		Retention time (min)
	Quantification	Confirmation	
Thymol	135	150; 91 and 115	15.819
Carvacrol	135	150; 91 and 107	16.002

### *In vitro* antioxidant activity of thyme tincture

The antioxidant activity of thyme tincture was evaluated by the DPPH free radical scavenging test and the result was presented by IC_50_ value, defined as the concentration of the antioxidant required to scavenge 50 % of DPPH present in the test solution. The results demonstrated that investigated tincture had a strong radical scavenging activity with an IC_50_ value of 0.169 mg/ml. A comparison was made with ascorbic acid (vitamin C), well-known potent antioxidant and free radical scavenger, which exhibited IC_50_ value of 0.642 μg/ml. According to that, 1 mg of ascorbic acid was found to be equivalent to 263.2 mg of the thyme tincture.

Antioxidant activity of thyme was reported to be derived mostly from the presence of phenolic compounds, particularly thymol and carvacrol. We measured total phenolic content in order to determine the extent of contribution of these chemical compounds to free radical scavenging capacity of thyme tincture. Total phenolic content of investigated tincture was measured using the Folin-Ciocalteu method and the high amount of total phenols (4859.77 mg GAE/l) was determined.

### Influence of thyme preparations on CCl_4_-induced serum biochemical parameters

Biochemical parameters, related to hepatic and renal function, were determined in serum of rats and the results are presented in Table 2. Cholesterol and triglycerides levels were examined in order to evaluate the effect of thyme preparations on metabolic function of the liver. Triglycerides concentrations were significantly decreased in serum of CCl_4_-treated animals. Thyme tincture and syrup could partially revert those values, but they were still significantly lower than those of saline-treated group. CCl_4_induced a slight decrease in cholesterol levels, which was partially recovered after administration of thyme tincture. The intake of thyme preparations alone did not affect significantly the metabolic function of the liver. Impaired excretory function of the liver was observed after treatment of animals with CCl_4_, which is indicated by significant increase in both total and direct bilirubin levels in serum, compared to the control group. Co-administration of thyme formulations partially recovered bilirubin levels, while administration of thyme tincture and syrup alone did not change significantly these values when compared to saline-treated group.

**Table 2:** Effects of thyme tincture and syrup on biochemical parameters in serum

	Con S	Con CCl_4_	Tin	Tin + CCl_4_	Syr	Syr + CCl_4_
Triglycerides (mmol/l)	1.31 ± 0.24	0.34 ± 0.05 ^a^	0.89 ± 0.16	0.51 ± 0.10 ^a^	1.29 ± 0.20*	0.59 ± 0.05 ^a^
Cholesterol (mmol/l)	1.88 ± 0.25	1.38 ± 0.06	1.87 ± 0.32	1.56 ± 0.07	1.95 ± 0.17	1.37 ± 0.12
Total bilirubin (μmol/l)	2.18 ± 0.10	3.08 ± 0.17 ^a^	2.33 ± 0.18	2.62 ± 0.31	1.98 ± 0.07*	2.52 ± 0.10
Direct bilirubin (μmol/l)	0.17 ± 0.02	0.58 ± 0.04 ^a^	0.37 ± 0.07	0.52 ± 0.12 ^a^	0.25 ± 0.06*	0.40 ± 0.03
AST (U/l)	127.3 ± 7.2	859.3 ± 160.3 ^a^	199.8 ± 46.3*	1430.7 ± 285.3 ^a^	120.0 ± 5.8*	626.7 ± 108.5
ALT (U/l)	47.3 ± 3.8	144.5 ± 32.2 ^a^	48.0 ± 3.5*	209.8 ± 38.5 ^a^	41.0 ± 1.9*	101.3 ± 16.3
Urea (mmol/l)	9.22 ± 0.44	10.47 ± 0.35	9.15 ± 1.07	10.75 ± 0.30	10.67 ± 0.47	8.95 ± 0.78
Creatinine (μmol/l)	45.50 ± 0.72	50.67 ± 1.12	45.00 ± 1.65*	51.33 ± 1.02 ^a^	44.17 ± 0.60*	50.17 ± 1.89

All values are expressed as mean ± SEM

^a^significantly different from Con S group; *significantly different from Con CCl_4_ group; *p* < 0.05

The extent of hepatocellular damage was assessed by measuring activities of AST and ALT in serum, which were increased 7- and 3-fold respectively in CCl_4_-treated group, compared to the control group. This effect was additionally exacerbated after the co-treatment with thyme tincture. On the contrary, co-administration of thyme syrup reduced the values of AST and ALT in serum, compared to CCl_4_-treated animals. Activities of these enzyme markers of liver injury were not significantly changed in animals treated only with thyme preparations compared to the control group.

Concentrations of urea and creatinine, as indicators of renal function, were also determined in serum. Nephrotoxic potential of CCl_4_ has been observed, since it induced a slight decrease in urea and creatinine levels, which were not significantly changed after co-treatment with thyme preparations. The intake of thyme tincture and syrup alone did not affect significantly renal excretory function.

### Influence of thyme preparations on CCl_4_-induced oxidative stress parameters in liver

**Fig. 2:**
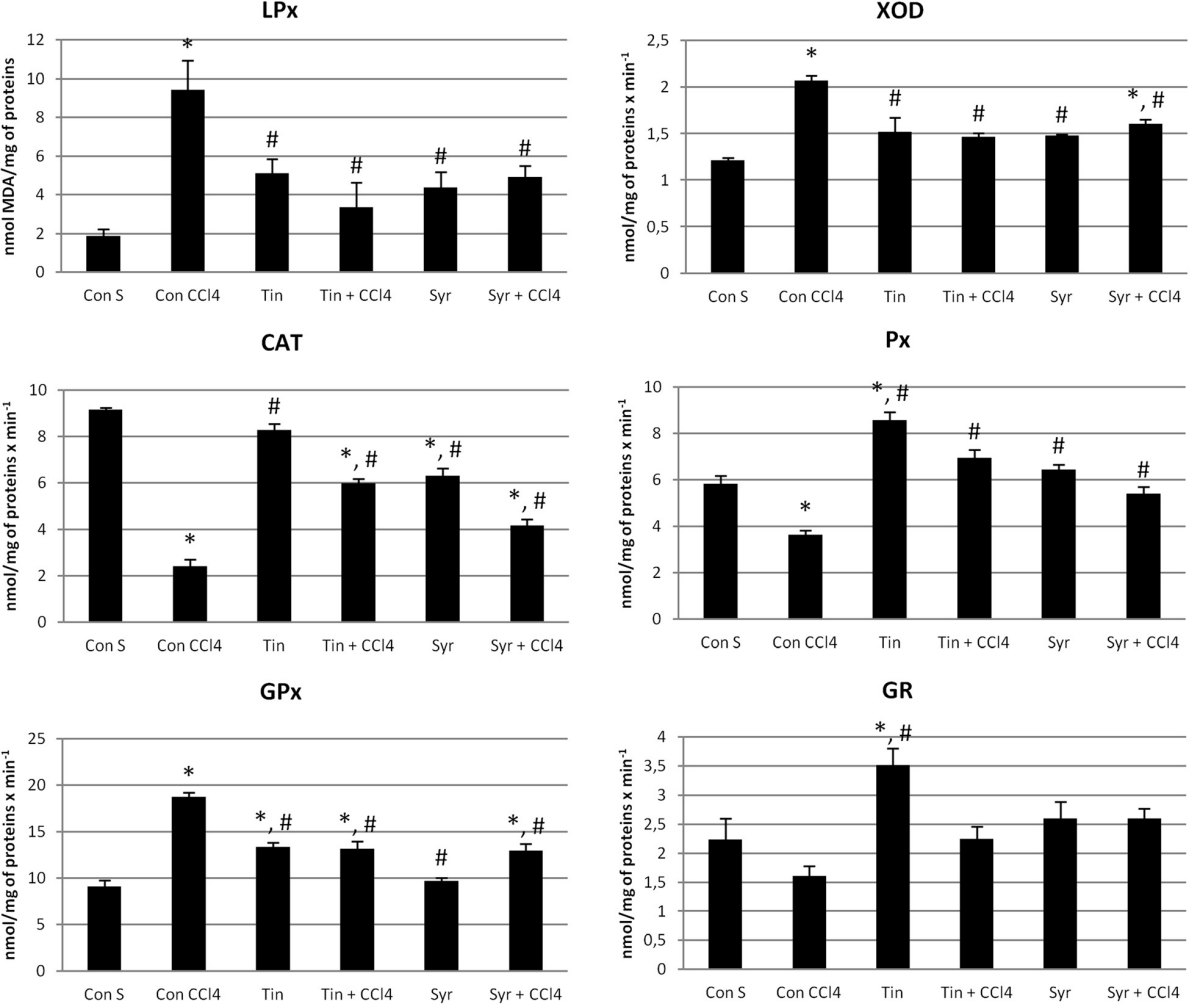
Levels of lipid peroxidation (LPx), xantine oxidase (XOD), catalase (CAT), peroxidase (Px), glutathione peroxidase (GPx), and glutathione reductase (GR) in liver homogenates. (*) significantly different from Con S group; (#) significantly different from Con CCl_4_ group; *p* < 0.05

In the present study, we aimed to evaluate the effects of thyme on CCl_4_-induced liver injury and to explore whether its mechanism of action is associated with modulation of hepatic oxidative status. Thus, we measured malondialdehyde (MDA) levels and activities of xanthine oxidase (XOD), catalase (CAT), peroxidase (Px), glutathione peroxidase (GPx), and glutathione reductase (GR), as biomarkers of the effects of studied thyme formulations, and the results are presented in Fig. 2.

The single dose of CCl_4_ induced 5-fold increase of hepatic MDA level, when compared to the control group. Co-treatment with both thyme tincture and syrup during 7 days succeeded to decrease MDA significantly in liver tissue, compared to CCl_4_-treated group. The intake of thyme preparations alone induced a slight increase in MDA levels, but it was not statistically significant. The activity of pro-oxidant XOD enzyme was changed in a similar manner after treatment of animals with CCl_4_ and thyme preparations. XOD activity was significantly elevated in CCl_4_-treated group, while co-treatment with thyme tincture and syrup could significantly reduce activity of this enzyme. In animals co-treated with thyme syrup, XOD activty remained significantly higher when compared to that of saline-treated group.

Oxidative stress induced by CCl_4_ resulted also in a modification of antioxidant enzymes activities in the liver. Activities of CAT, Px, and GR were significantly reduced in CCl_4_-treated animals, compared to the control group, while GPx showed significantly increased activity in the liver. Co-administration of thyme preparations restored activities of CAT and Px towards normal values, and these increases were more pronounced in the group co-treated with tincture than with syrup. Thyme preparations significantly reduced the activity of GPx when applied with CCl_4_ in comparison to animals treated only with CCl_4_, and the tincture and syrup exerted these effects in a similar manner. On the other hand, the intake of thyme tincture alone resulted in significant increase of GPx, while syrup did not have that effect. Thyme tincture, when applied alone, also induced significant elevation of hepatic GR activity in comparison to both saline- and CCl_4_-treated animals, while that activity was not significantly changed in other studied groups.

### Influence of thyme preparations on liver histology

**Fig. 3:**
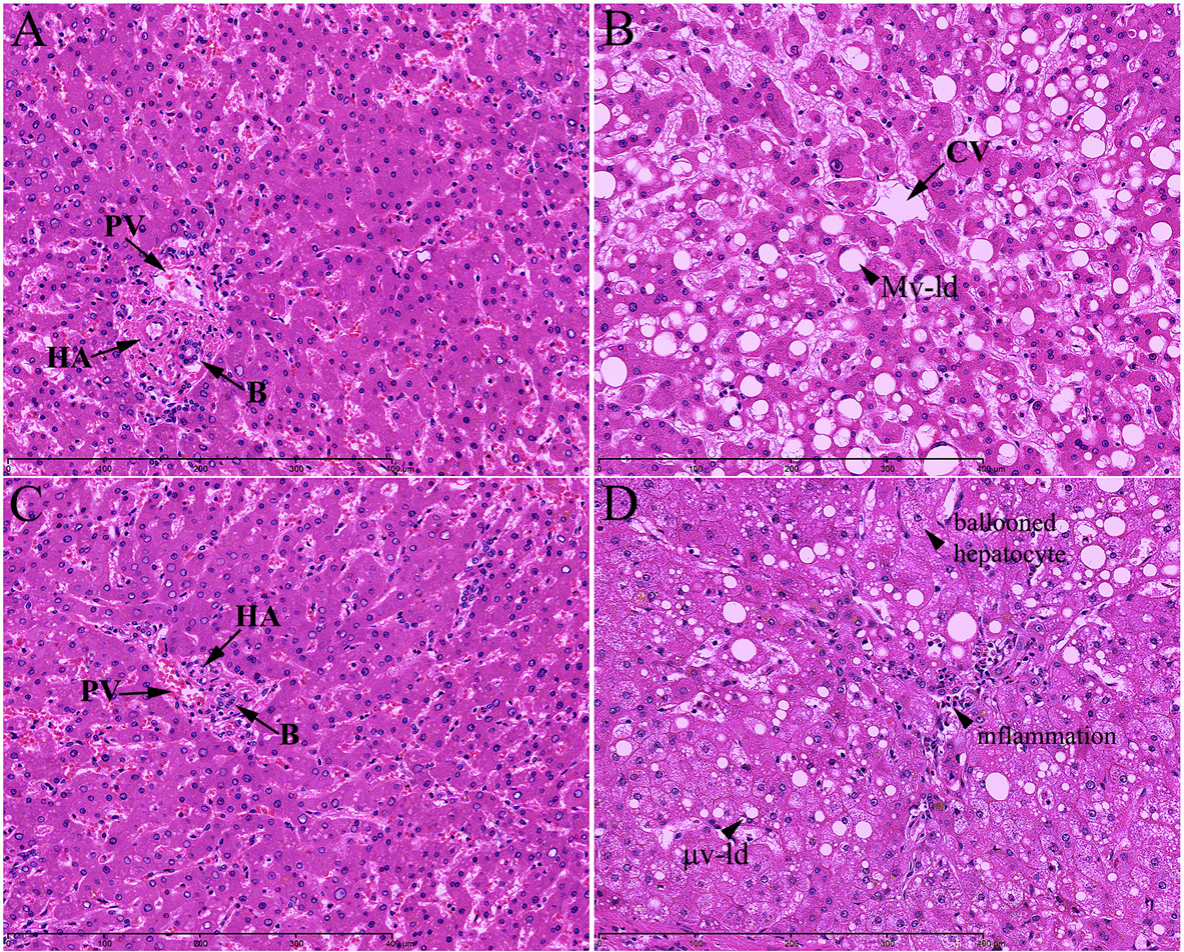
Photomicrographs of the liver sections stained with standard H&E technique taken at the magnification of 200x. **a** sections from control animals (portal triad elements, PV—portal venule, HA—hepatic arteriole, B—bile ductule); **b** animals treated with CCl_4_ alone (C—central vein, Mv-ld—macrovesicular lipid droplet); **c** thyme tincture alone (portal triad elements, PV—portal venule, HA—hepatic arteriole, B—bile ductule); and **d** sections from animals treated with both CCl_4_ and thyme tincture (μv-ld—microvesicular lipid droplet, ballooned hepatocytes, inflammation in portal tracts)

The general liver morphological changes were examined by standard H&E staining. Histopathological examination of liver sections of control group showed normal cellular architecture with distinct hepatic cells, sinusoidal spaces and central vein. Hepatocytes were well formed with homogeneous cytoplasm and regular sized, centrally located nuclei. The sinusoidal capillaries were normal-sized without lymphocytic infiltrate. Hepatic artery, portal vein and bile ducts could be observed in portal spaces (Fig. 3a). Seven-day treatment of rats with thyme tincture resulted in non-observable changes of liver histology in comparison to saline-treated animals (Fig. 3c).

Histopathological studies of liver sections of CCl_4_-intoxicated rats, alone and in combination with thyme tincture, showed similar histopathological findings of steatosis or steatohepatitis. Most of hepatocytes contain lipid droplet that may be either macrovesicular or microvesicular (Fig. 3b,d). In some cases mild inflammation was present in portal spaces, while numerous hepatocytes are ballooned. These changes in the hepatic architecture were not observed in any of the control rats or animals treated with thyme tincture alone. Thyme co-treatment could not improve CCl_4_-induced morphological changes, since no significant histological differences were observed between rats treated with CCl_4_ alone and in combination with thyme tincture (Fig. 3d).

## Discussion

In the present study, we evaluated antioxidant activity of thyme preparations and their influence on hepatic function using the hepatotoxicity model induced by CCl_4_. The concentrations of main active constituents of thyme, thymol and carvacrol, were determined, and thymol was found to be predominant, with approximately 16 times higher concentration in comparison to carvacrol, in both tincture and syrup.

Antioxidant activity of thyme and its extracts is dependent mainly on essential oil content and composition. It was demonstrated that phenolic chemotype of thyme had a stronger antioxidant potential than a non-phenolic chemotype, and that thymol and carvacrol mostly contributed to the antioxidant activity of thyme. Oxidation products of thymol, such as thymoquinone and thymohydroquinone, are even more potent antioxidants than thymol, as tested with several different methods [8, 37].

Many *in vitro* studies confirmed antioxidant properties of thyme extracts. It was shown that thyme extract had stronger scavenging activity towards both DPPH and ABTS radicals and higher total phenolic content, in comparison to sage and stinging nettle [38]. Similiarly, extracts of thyme, sage, and marjoram prepared using methanol, ethanol, diethyl ether and hexane were examined using DPPH assay, and methanolic thyme extract had the lowest IC_50_ value, i.e. exhibited the strongest antioxidant capacity [39]. Tinctures of eleven plants used as spices, including thyme, were tested for their antiradical properties by means of the DPPH assay over a 2 year period, and thyme exhibited stable antioxidant activity indicated by little variation in scavenging activity after 1 year [40].

The phenolic concentrations in ethanolic thyme extracts in different studies, calculated as mg GAE per gram of dry weight ranged from 3.36 [41] to 7.30 [39]. In our study, high amount of total phenols of 4859.77 mg GAE/l was measured, and that is in accordance with the results of the study of Kulišić et al., 2006 [42] who determined total phenolic content of 2000 mg GAE/l and 4400 mg GAE/l in aqueous tea infusions obtained from thyme and wild thyme, respectively. The discrepancy between results of different studies can be attributed to several factors, including geographical location of the plant, parts of plants used for extracts preparation, extraction protocol, such as solvent, temperature, and time [10].

The influence of thyme preparations on CCl_4_-induced liver injury was determined by assessing serum levels of aminotransferases (AST and ALT). In our study, hepatotoxicity induced by CCl_4_ was reflected by a marked elevation of AST and ALT activities, but also by notable histopathologic alterations. Significant elevation of ALT and AST in serum after administration of CCl_4_ indicates a loss of the structural and functional integrity of the liver, and the release of these cytoplasmic enzymes into systemic circulation. In addition, our results reported increased level of serum bilirubin, thereby suggesting impaired excretory function of the liver. This is in agreement with the results of many earlier studies using a model of CCl_4_-induced liver damage. In this work, necrosis of hepatocytes was confirmed histologically.

Administration of thyme preparations alone did not change significantly biochemical nor histological markers of hepatic function, when compared to saline-treated group. On the other hand, co-administration of thyme tincture resulted in unexpected exacerbation of AST and ALT values in serum, while thyme syrup managed to reduce activites of aminotransferases, in comparison to CCl_4_-treated animals. Opposing effects of thyme tincture and syrup on the liver suggest that the dose of tincture is too high, since it is known that herbal polyphenols in high concentrations and under some circumstances such as the presence of transition metals or peroxidases, can become pro-oxidants and cause cytotoxicity and liver damage through generation of ROS [43]. Besides, thymol and carvacrol, as main active constituents of thyme, are monoterpenes, which principally do not exhibit dose-dependent effects and for which is necessary to find the most appropriate dose range that shows effectiveness [44].

Previous studies demonstrated that oral administration of a concentrated ethanolic extract of thyme in subacute toxicity tests resulted in increased weights of liver in mice [6]. Furthermore, in the study of Liu et al., 2011 [45], 16 phenolics and five botanical extracts, including methanolic thyme extract, were evaluated in both a human and a rat hepatocyte cell line using a battery of eight endpoints, covering a variety of biological activities relevant to hepatotoxicity. Cluster analysis was used to group the phenolics and extracts, and thyme extract clustered in the presumptive hepatotoxic group in rat MH1C1 hepatoma cells, but not in human HepG2/C3A cells. On the contrary, the hepatoprotective effects of thyme have been observed in experimental models of liver injury induced by paracetamol [24], NDEA [21], and aflatoxins [20]. Likewise, dietary supplementation of thyme extract to rats during 8 weeks was able to ameliorate the acute hepatotoxicity caused by CCl_4_ [22]. The results of our study differ from these results, and that can be explained by differences in applied doses and treatment periods. Hepatoprotective activity has been also demonstrated for main thyme constituents. Thymol ameliorated CCl_4_-induced liver injury in mice and it was suggested that it suppressed cytochrome P450 mediated metabolic activation of CCl_4_ [46]. Furthermore, carvacrol exerted antioxidant and hepatoprotective effects in a model of D-galactosamine-induced hepatotoxicity in rats [47].

Promising results in terms of antidiabetic activity of thyme have been observed in both streptozotocin [13] and alloxan models [48] of diabetes mellitus in rats, suggesting its potential use in the treatment of metabolic syndrome. The intake of thyme extract managed to reduce serum concentrations of cholesterol and triglycerides, but we did not observe these effects. Seven-day treatment of rats with thyme tincture resulted in reduction of triglycerides levels in serum, but it was not statistically significant.

In addition to determination of hepatic function, the influence of thyme preparations on renal function has been also investigated in our study. CCl_4_ induced a slight decrease in urea and creatinine levels in serum of rats. In our previous studies [49, 50], CCl_4_ was applied in a higher dose (1 ml/kg) that exerted more pronounced nephrotoxic effects indicated by significant increase of urea and creatinine concentrations in serum. The parameters of renal function were not significantly changed after co-treatment with thyme preparations, which is in accordance with results of the study of Ozkol et al., 2013 [13]. On the other hand, significant nephroprotective activity was demonstrated for thyme in aflatoxin-intoxicated rats [51].

Considering the role of oxidative stress in etiopathogenesis of liver injury induced by most xenobiotics, we evaluated the influence of pharmaceutical formulations containing thyme on lipid peroxidation and endogenous antioxidant defense system in liver. We used the model of acute liver damage induced by CCl_4_, since this compound is metabolized via reductive dehalogenation involving cytochrome P450, resulting in formation of ROS that cause the peroxidation of membrane lipids. Administration of CCl_4_ induced significant elevation of hepatic levels of MDA, a major secondary product of lipid peroxidation and indicator of cell membrane injury (Fig. 2). Co-administration of thyme preparations managed to prevent CCl_4_-induced increase of MDA, suggesting their ability to preserve cellular integrity. Similar to our results, it was shown that dietary supplementation of thyme extract and essential oil during 8 weeks was able to ameliorate CCl_4_-induced lipid peroxidation in liver of rats [22]. CCl_4_ also increased the activity of pro-oxidant enzyme XOD that forms superoxide anion radical and hydrogen peroxide, and the intake of thyme significantly reversed these activities towards normal values.

The activities of antioxidant enzymes were also significantly changed after administration of CCl_4_ (Fig. 2). This toxicant induced a significant reduction of CAT, Px and GR activities, which can be explained by their inactivation induced by excessive production of free radicals. On the other hand, activity of GPx was increased in the liver after administration of CCl_4_. Similar results were obtained in our previous study [49] and the study of Hsiao et al., 2003 [52], where the increase of GPx activity and reduction of CAT activity were demonstrated after CCl_4_ treatment. It is well known that CAT has an important role in the elimination of ROS derived from the redox process of xenobiotics in the liver, and that it is easily inactivated by lipid peroxides. On the other hand, under oxidative stress conditions, GPx can be upregulated as the adaptive response of the hepatocytes to oxidative damage and its activity can be increased [53]. In several studies [20, 22], all antioxidant enzymes were decreased after application of CCl_4_, but these differences may be attributed to different treatment protocols.

Antioxidant activity of thyme has been ascertained in several *in vivo* models, beside models of liver damage. The essential oil and particularly aqueous tea infusion obtained from thyme exerted a protective role on copper-induced LDL oxidation [54]. Furthermore, aqueous extracts of thyme could prevent inhibition of glucose-6-phosphate dehydrogenase activity in erythrocytes and subsequent hemolysis caused by nitrosative stress [55]. Several studies investigated the influence of thyme on antioxidant status, including activities of antioxidant enzymes. It has been demonstrated that rats whose diets were supplemented with thyme oil retained higher antioxidant capacity during their life span by preventing the unfavourable age-related decline in activities of SOD in the liver and heart of old rats [56]. Likewise, oral administration of ethanolic extract of thyme enhanced plasma antioxidant status in healthy rats, and it was also shown that protection against oxidative stress by thyme occured primarily through direct antioxidant effects of its main constituents, but that it may be also related to the phenolic metabolites, such as thymol sulfate [57].

The results of our study have determined that thyme mediates its antioxidant activity through both direct free radical scavenging and activation of physiological defense mechanisms. Despite demonstrated antioxidant activity, thyme preparations unexpectedly could not ameliorate liver injury in rats. The role of oxidative stress in liver injuries is highly significant, but it should be emphasized that several other pathophysiological mechanisms of drug-induced hepatotoxicity have been shown to be involved, including inflammatory processes. Although anti-inflammatory effects of thyme have been demonstrated *in vivo*, the isolated active compounds thymol and carvacrol have shown antagonistic effects. It is known that thymol may have an irritative effect that likely involves histamine and prostanoids, contrary to carvacrol with established anti-inflammatory activity [11]. Given that thymol was found to be the predominant active compound in investigated thyme preparations in our study, the role of inflammatory processes may explain our results, although a more in-depth evaluation of the mechanisms involved should be examined.

## Conclusions

Pharmaceutical formulations containing thyme may aggravate existing hepatotoxicity, probably through involvement in cellular inflammatory processes. The lack of clinical safety and toxicity data for thyme, and many other herbs that are increasingly being used, suggests the necessity of further investigations regarding their influence on hepatic function, including the evaluation of molecular mechanisms involved in order to exploit them for potential therapeutic benefits.
